# The association of malaria infection and gestational hypertension in Africa: Systematic review and meta-analysis

**DOI:** 10.7189/jogh.10.020417

**Published:** 2020-12

**Authors:** Henry A Mruma, Ruth McQuillan, John Norrie

**Affiliations:** 1 Muhimbili University of Health and Allied Sciences (MUHAS), Dar es Salaam, Tanzania; 2 Usher Institute, The University of Edinburgh, Edinburgh, UK

## Abstract

**Background:**

The World Health Organisation (WHO) estimates that hypertensive disorders of pregnancy (HDP) contribute 14% to global maternal mortality. HDP encompasses several subcategories, including gestational hypertension (GH) and pre-eclampsia. These two conditions are both characterised by a rise in blood pressure, with an onset from 20 weeks of gestation. They also share some common risk factors. The current definition of pre-eclampsia includes raised blood pressure in the absence of proteinuria, thus presenting the two conditions as a spectrum. In this article, we refer to both conditions as gestational hypertension, which is our outcome of interest. The aetiology of GH is not yet clearly understood. Observational studies have suggested that malaria may be associated with GH. However, the evidence from these small studies has been inconclusive. Having a better understanding of the association between malaria and GH may help inform prevention strategies to reduce maternal and infant mortality and morbidity.

**Methods:**

In assessing the association between malaria infection and GH we explored open access articles published in the English language on Medline, Embase, WHO GIM and Google scholar. The subject related articles were retrieved and processed according to preferred reporting items in systematic reviews and meta-analyses (PRISMA) guidelines. Search date was 9th week of 2018. Inverse variance weighting method in Revman 5 software (Cochrane Collaboration, London, United Kingdom) was used to aggregate evidence by computing the pooled odds ratio to show the nature and strength of the relationship between malaria and GH.

**Results:**

Using critical appraisal skills program (CASP) checklist tool we identified four good quality case-control studies. The total sample size was 1281 women out of which 518 were cases. These studies together show malaria is associated with GH with an overall odds ratio of 2.67, 95% confidence interval (CI) = 1.58-4.53. Heterogeneity of the individual studies supported fixed effect modelling assumptions (I^2^ = 0%). Malaria infection may have a constant effect on GH across different African populations. The funnel plot did not suggest publication bias however, the four studies involved in the meta-analysis were insufficient to rule it out.

**Conclusions:**

Our findings provide evidence of an association between malaria infection and gestational hypertension; this underscores the need to control malaria especially during pregnancy.

Malaria is a protozoan blood infection that primarily affects and destroys red blood cells (RBC) thus causing a release of endotoxin that triggers an inflammatory reaction [1]. Malaria is a common disease in tropical countries that claims the lives of many children [2]. It also preferentially affects women during pregnancy resulting in maternal complications and mortality [3,4].

Pre-eclampsia is one of the subcategories of hypertensive disorder of pregnancy (HDP). Its aetiology is not well understood. WHO estimates that HDP accounts for 14% of global maternal mortality [5]. Pre-eclampsia is defined by two main features: raised blood pressure and proteinuria with an onset on or after 20 weeks of gestation [6]. A dysfunctional placenta is the basis of most theories for the cause of pre-eclampsia [7]. To date, most studies on malaria and pre-eclampsia have focused on the impact of malaria on the placenta. Several studies suggest that placental malaria infection is associated with pre-eclampsia and gestational hypertension (GH), the hypothesised mechanism is that of a dysfunctional placenta affected by malaria parasites [8-10].

Gestational hypertension is another subcategory of HDP. It is characterised by the increase in blood pressure with an onset of 20 weeks onwards without proteinuria [6]. Thus, proteinuria is the main distinguishing feature between these two subcategories. Lately, the definition of pre-eclampsia has been revised to include situation were pre-eclampsia occurs in the absence of proteinuria [6,11]. This presents the view that these conditions are a spectrum rather than discrete entities. Studies have also shown that these two conditions share a number of preconception cardiovascular risk factors [12]. The two conditions have also been found to have a similar association with the risk of cardiovascular disease in later life [12].

Recently, a new mechanism has been proposed, it hypothesises that malaria infection causes hypertension via inflammatory reaction on the endothelial lining in blood vessels. It is hypothesised that inflammatory processes on the endothelium due to malaria infection may play a role in causing hypertension [1]. Studies in Asia that examined maternal serum iron and ferritin have suggested oxidative stress caused by free iron radicals may play a role in causing pre-eclampsia [13,14]. Thus, the inflammatory and oxidative stress due to malaria toxin and RBC destruction may lead to pre-eclampsia. We also know that malaria infection on its own may induce proteinuria [15]. Therefore, an alternative pathway mechanism may exist where malaria infection during pregnancy may be causing both hypertension and proteinuria, which are the main features that define pre-eclampsia, without placental pathway involvement. It is also possible that malaria infection will only induce hypertension without proteinuria to resemble GH.

In this regard, it is worth examining the association of malaria and hypertension during pregnancy (with or without proteinuria) to identify its total effect on the burden of HDP. From this point onwards in our article, we have regarded GH to imply the combination of both, pre-eclampsia and gestational hypertension proper. For this reason, our systematic review and meta-analysis regards our outcome of GH to be with proteinuria (pre-eclampsia) or without.

Earlier studies had suggested an association between malaria infection in the rainy season and increased maternal mortality due to pre-eclampsia [16]. Women are prone to develop malaria throughout pregnancy, but the peak period seems to occur just before the onset of pre-eclampsia [3,17]. The peak incidence of malaria during pregnancy has been shown to occur at 13-16 weeks of gestation, which is prior to the onset of the GH which commences at 20 weeks of gestation onwards [3,17]. This sequential timing of the malaria risk factor and the occurrence of the disease suggest the natural existence of a temporal relationship between the risk and the outcome.

Malaria infection could be an important contributing factor to GH in tropical countries where malaria is prevalent. About 25 million pregnant women from Sub Saharan Africa are at risk of malaria infection every year, while approximately 25% have been found with evidence of placental malaria infection at delivery [18]. Understanding the association of GH with malaria infection can inform prevention strategies especially in malaria endemic regions. Even a weak association found between malaria and GH will likely have a high population attributable risk, due to the high prevalence of malaria in these settings. We set out to conduct a systematic review and meta-analysis with the aim to aggregate available evidence on this relationship. Evidence on the association between malaria infection and GH is likely to attract further studies to explore causality.

## METHODS

We explored on our research question on the association of malaria infection and GH by searching articles on MEDLINE, WHO Global Index Medicus (GIM), Google scholar, EMBASE databases. We extended the search to include grey literature and hand searching of referenced studies.

### Search terms/strategy

We used these search terms: Malaria, Placental malaria, Hypertension-pregnancy induced (pre-eclampsia, eclampsia, HEELP). Exposure keywords (malaria or placental malaria) were combined using the Boolean word AND with outcome keywords (pregnancy induced hypertension, pre-eclampsia). These keywords were modified to fit the respective databases.

### Search criteria

We restricted our search to full articles, English language and human studies with no limits on the year of study. We included observational studies (case-control, cross-sectional and cohort) that explored the association between malaria and GH, and excluded case reports, case series and ecological studies. We also excluded studies that did not ascertain the diagnosis of malaria by laboratory tests. Our final analysis only included final studies that had measured the association between malaria infection and GH while adjusting for confounding factors.

### Study population, case, and outcome definition

Our study population was all pregnant women, the exposure was malaria infection during pregnancy, ie, women diagnosed with malaria during pregnancy, or with post-pregnancy diagnosis of placental malaria. Malaria Infection was diagnosed clinically by signs and symptoms accompanied by a blood test (rapid blood test for malaria or blood slide for microscopy). Malaria infection during pregnancy can invade the placenta to cause placental malaria, which can be confirmed after delivery by examining the placenta tissue. In this study, we considered either malaria infection during pregnancy or placental malaria confirmed after delivery as our exposure of interest. Women free of malaria diagnosis during pregnancy or free from post-pregnancy diagnosis of placental malaria were regarded as the unexposed group. The outcome of interest was women with a diagnosis of GH.

### Data extraction

The searched papers from the databases were downloaded and managed on Endnote X7 software (Clarivate Analytics, Philadelphia, PA, USA). Duplicates were removed by the software and followed by manual search and removal of duplicates. Titles and abstracts of identified studies were screened for relevance. Those not considered relevant were excluded.

Additional papers were searched from grey literature sources such as relevant institutions’ repositories (eg, universities). Reference lists of the selected papers were examined to identify additional papers.

We extracted key information from the articles; author, year, study design, country of study, sample size and univariate odds ratios (OR) and confidence interval (unadjusted). Where it was available, multivariable (adjusted) odds ratios and confidence intervals were extracted together with the list of variables used in the adjusted model.

### Quality appraisal

A full text review of the selected articles was done to select the eligible articles based on our inclusion and exclusion criteria. Critical appraisal skills programme (CASP) checklist tools were used to guide the quality assessment of the observational studies [19].

We reduced misclassification bias of the exposure on malaria infection by including only those studies which had ascertained the diagnosis of malaria with a laboratory test.

### Analysis

**Figure 1:**
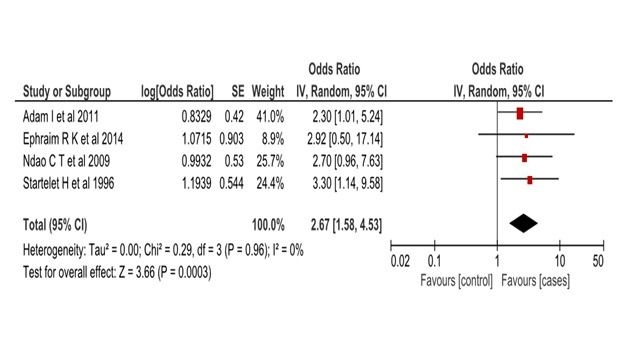
Forest plot of the meta-analyzed studies. This forest plot shows the odds ratio of the individual studies and their pooled effect in the association between malaria infection and GH. The individual studies are weighed by the inverse variance method. The overall odds ratio is 2.67 with 95% CI = 1.58-4.53. The Heterogeneity of the studies is given by I^2^ = 0%. Suggesting strong homogeneity in their results.

**Figure 2:**
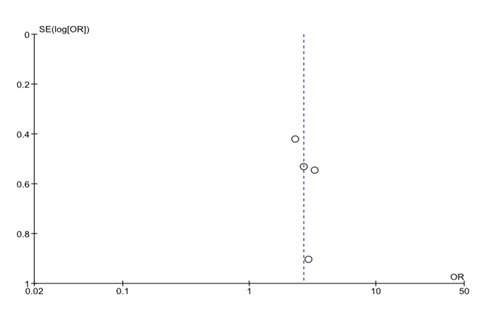
Funnel plot of the meta-analyzed studies. This funnel plot shows that studies with large and small variance had similar estimates of the effect of malaria infection on GH.

We assessed the heterogeneity of the included papers to determine the feasibility of a meta-analysis. We had pre-set to accommodate moderate heterogeneity (I^2^ = 30%) due to the expected population variability of the exposure outcome association. We had anticipated the association between malaria and GH to vary widely across study populations hence we used the random effects model to estimate the pooled effect in the meta-analysis. Inverse variance weighting was used to compute the overall odds ratio in Revman 5 software (Cochrane Collaboration, London, United Kingdom). A forest plot was generated to display overall study results (**Figure 1**). We used a funnel plot to assess potential publication bias of the selected papers (**Figure 2**).

## RESULTS

### Quality appraisal

The results of our search are shown graphically in the preferred reporting items for systematic reviews and meta-analyses (PRISMA) diagram (Figure S3 in the **Online Supplementary Document**). After the removal of duplicates, our search identified 470 papers, of which 462 were excluded based on screening titles and abstracts. CASP tools were used for a quality appraisal [19]. Eight papers were assessed for eligibility only four were suitable for the meta-analysis (Table S2 in the **Online Supplementary Document**). The remaining four papers were excluded because; one study had a different outcome group. ie, women with all types of hypertensive disorders of pregnancy, thus included women with chronic hypertension. The other three studies did not present the association of malaria infection and GH, adjusted by other factors.

### Characteristics of the studies

The first included study was a hospital-based study from Senegal, a case-control with 223 cases and 240 controls, who were matched by age, parity and prematurity. Their results from a multivariable logistic regression showed an odds ratio of 2.7, 95% confidence interval (CI) = 0.9-7.6 after adjusting for placenta malaria, residence, parity, past pregnancies, antenatal visit, family history of hypertension, the period of delivery-seasonality, illiteracy and marital status [20].

The second study was also a hospital-based study from Senegal, with 32 cases and 220 controls, who were similar in mean age and number of previous pregnancies. Their results from a multivariable logistic regression showed an odds ratio of 3.3, 95% CI = 1.1-9.5 after adjusting for age, no of previous pregnancies, twin delivery, maternity centre and date of delivery [21].

The third study was a hospital-based case-control study from Ghana, with 120 cases and 160 control. They excluded chronic hypertension, patients on antihypertensive drugs, eclampsia, diabetes, autoimmune and renal diseases. Their multivariable logistic regression showed an odds ratio of 2.9, 95% CI = 0.5-17 after adjusting for age, gravidity, parity, body mass index (BMI), contraceptive use, abortion, new paternity and Malaria [22].

The above three studies are from West Africa, the fourth study was a hospital-based case-control from Sudan, north Africa. It recruited 143 cases and 143 controls while excluding twins and diabetes. Its results showed an odds ratio of 2.3, 95% CI = 1.0-5.2 after adjusting for age, primigravidae, history of malaria, family history of hypertension, BMI, blood group, placenta malaria, education level and lack of antenatal clinic visit [9].

The four small studies (Table S1 in the **Online Supplementary Document**) did have wide confidence intervals which suggest their imprecise estimate, but upon aggregating them the pooled estimate resulted in a marrow confidence interval (OR = 2.6, 95% CI = 1.5-4.5).

The four studies used a similar cut-off levels for hypertension, ≥140mmHg systolic blood pressure and ≥90mmHg diastolic blood pressure. They also adjusted for common confounding variables; mother’s age, history of hypertension and number of previous pregnancies (gravidity). BMI and diabetes status were adjusted in two studies. However, there were some variation in the cut-off point for proteinuria, ≥2+, >2 and≥“1+” in Adam et al, Ndao et al, and Ephraim et al studies respectively. These changes in cut-offs that have evolved over time could result in misclassification in the control groups, an effect likely to dilute the strength of observed association. Therefore, the true pooled point estimates from these studies could be on the higher side of our pooled confidence interval (OR = 2.6, 95% CI = 1.5-4.5).

## DISCUSSION

The relationship between malaria infection and GH has long been explored by different small studies in Africa [16,21]. Our meta-analysis has been able to show an overall statistically significant association between malaria infection and GH, odds ratio 2.67 (95% CI = 1.58-4.53). The common variables adjusted in all the four studies were; mother’s age, gravidity and history of hypertension. The exposure of interest was placenta malaria or malaria infection during pregnancy while our outcome of interest was either a diagnosis of gestational hypertension with proteinuria (pre-eclampsia) or without proteinuria. Malaria infection is a common infection in tropical countries, especially during pregnancy [18]. This result suggests that it may be an important risk factor for GH in the region.

The strength of our paper is that it employed a wide search strategy in different databases and used appropriate quality assessment methods. CASP checklist tool was adopted to evaluate the quality of primary studies. The selected papers had a focused research question to explore the quantitative relationship between malaria infection and GH. They had similar definitions of the exposure and outcome in line to our defined exposure and outcome definitions. These studies had adjusted for possible confounders through restriction, matching and analysis. Maternal age, parity and cardiometabolic factors such as diabetes, BMI, chronic hypertension, were adjusted in the analysis. This adjustment has enabled to observe the nature of the relationship, which seems to be obscured by the strong confounding factors in studies that only rely on univariable analysis. Thus, the aggregate result from our meta-analysis gives a credible result on the nature of the overall association.

The measure of heterogeneity I^2^ was 0%, this was different from our initial assumptions. We had assumed the relationship between malaria and GH to vary widely across societies and thus adopted the random effects model, which often produces a higher measure of heterogeneity. Our findings appear to suggest that there is a fixed effect of malaria on GH across study populations. Three of our analyzed studies were from West Africa and one from Sudan. However, our analysis had few studies that may have produced the low I^2^ values. Therefore, our results show the studies are homogenous and hence allow us to meta-analyze them.

Although our analysis had few studies, the funnel plot seems to suggest that studies with high variability in their standard errors produced a similar effect size to studies with less variability. There is no asymmetry on the plot, a feature that indicates publication bias is unlikely, and all the four studies are centred close to the common effect (Figure S2 in the **Online Supplementary Document** ). This observation may be due to chance or absence of enough published papers from diverse populations. Although, the results do not suggest the presence of publication bias they evidence from the four paper is not sufficient to rule out publication bias.

The evidence from these studies is consistent with evidence from ecological studies that suggest an association between an increase in death due to pre-eclampsia during the rainy malaria seasons [16,23]. Dorman et al [24] also showed that malaria infection affected the uterine blood flow, a finding which is also consistent with our findings. Placenta dysfunction has been one of the theories linking malaria to pre-eclampsia [10].

This meta-analysis has aggregated evidence from a number of small studies that examined the association between malaria and GH. Our results strengthen the evidence for such an association. Our findings have potential policy implications on disease prevention, as it reinforces the importance of malaria prevention during pregnancy. Reducing malaria during pregnancy may have wider benefit beyond that of reducing the direct effects of malaria on the mother and the unborn baby. Molecular studies exploring this relationship could improve our understanding and identify intervention point on the theorised causal pathways.

## CONCLUSION

Malaria infection is associated with GH in tropical countries. Malaria also appears to exert a constant effect across the studied populations. Enhancing malaria control efforts on pregnant women could have direct benefits during pregnancy and avert long-term cardiovascular effects on mothers. Molecular studies are needed to further explore the causal mechanism.

## Additional material

Online Supplementary Document

## References

[1] Verdecchia P Angeli F Reboldi G Does malaria cause hypertension? Circ Res. 2016;119:7-9. 10.1161/CIRCRESAHA.116.309013 27340264

[2] Liu L Oza S Hogan D Chu Y Perin J Zhu J Global, regional, and national causes of under-5 mortality in 2000–15: an updated systematic analysis with implications for the Sustainable Development Goals. Lancet. 2016;388:3027-35. 10.1016/S0140-6736(16)31593-8 27839855 PMC5161777

[3] Brabin BJ An analysis of malaria in pregnancy in Africa. Bull World Health Organ. 1983;61:1005. 6370484 PMC2536236

[4] WHO. World Malaria Report. Geneva: 2019.

[5] Say L Chou D Gemmill A Tunçalp Ö Moller AB Daniels J Global causes of maternal death: a WHO systematic analysis. Lancet Glob Health. 2014;2:e323-33. 10.1016/S2214-109X(14)70227-X 25103301

[6] Brown MA Magee LA Kenny LC Karumanchi SA McCarthy FP Saito S The hypertensive disorders of pregnancy: ISSHP classification, diagnosis & management recommendations for international practice. Pregnancy Hypertens. 2018;13:291-310. 10.1016/j.preghy.2018.05.004 29803330

[7] Ahmed A Ramma W Unravelling the theories of pre- eclampsia: are the protective pathways the new paradigm? Br J Pharmacol. 2015;172:1574-86. 10.1111/bph.12977 25303561 PMC4354257

[8] Ndao CT Dumont A Fievet N Doucoure S Gaye A Lehesran JY Placental Malarial Infection as a Risk Factor for Hypertensive Disorders During Pregnancy in Africa: A Case-Control Study in an Urban Area of Senegal, West Africa. Am J Epidemiol. 2009;170:847-53. 10.1093/aje/kwp207 19679749

[9] Adam I Elhassan EM Mohmmed AA Salih MM Elbashir MI Malaria and pre-eclampsia in an area with unstable malaria transmission in Central Sudan. Malar J. 2011;10:258. 10.1186/1475-2875-10-258 21899731 PMC3224261

[10] Kidima WB Syncytiotrophoblast Functions and Fetal Growth Restriction during Placental Malaria: Updates and Implication for Future Interventions. BioMed Res Int. 2015;2015:451735. 10.1155/2015/451735 26587536 PMC4637467

[11] Tranquilli AL Dekker G Magee L Roberts J Sibai BM Steyn W The classification, diagnosis and management of the hypertensive disorders of pregnancy: A revised statement from the ISSHP. Pregnancy Hypertens. 2014;4:97-104. 10.1016/j.preghy.2014.02.001 26104417

[12] Egeland GM Klungsoyr K Oyen N Tell GS Naess O Skjaerven R Preconception Cardiovascular Risk Factor Differences Between Gestational Hypertension and Preeclampsia Cohort Norway Study. Hypertension. 2016;67:1173. 10.1161/HYPERTENSIONAHA.116.07099 27113053 PMC4861703

[13] Rayman MP Barlis J Evans RW Redman CWG King LJ Abnormal iron parameters in the pregnancy syndrome preeclampsia. Am J Obstet Gynecol. 2002;187:412-8. 10.1067/mob.2002.123895 12193935

[14] Zafar T Iqbal Z. Iron status in preeclampsia. Profess Med J. 2008;1:74-80.

[15] Ehrich JH Horstmann RD Origin of proteinuria in human malaria. Trop Med Parasitol. 1985;36:39-42. 3890120

[16] Etard JF Kodio B Ronsmans C Seasonal variation in direct obstetric mortality in rural Senegal: Role of malaria? Am J Trop Med Hyg. 2003;68:503-4. 10.4269/ajtmh.2003.68.503 12875305

[17] Moore KA Simpson JA Wiladphaingern J Min AM Pimanpanarak M Paw MK Influence of the number and timing of malaria episodes during pregnancy on prematurity and small-for-gestational-age in an area of low transmission. BMC Med. 2017;15:117. 10.1186/s12916-017-0877-6 28633672 PMC5479010

[18] Desai M ter Kuile FO Nosten F McGready R Asamoa K Brabin B Epidemiology and burden of malaria in pregnancy. Lancet Infect Dis. 2007;7:93-104. 10.1016/S1473-3099(07)70021-X 17251080

[19] CASP. Critical Appraisal Skills Programme Checklist. 2018. Available: https://casp-uk.net/casp-tools-checklists/. Accessed: 8 July 2020.

[20] Ndao CT Dumont A Fievet N Doucoure S Gaye A Lehesran JY Placental Malarial Infection as a Risk Factor for Hypertensive Disorders During Pregnancy in Africa: A Case-Control Study in an Urban Area of Senegal, West Africa. Am J Epidemiol. 2009;170:847-53. 10.1093/aje/kwp207 19679749

[21] Sartelet H Rogier C Milko-Sartelet I Angel G Michel G Malaria associated pre-eclampsia in Senegal. Lancet. 1996;347:1121. 10.1016/S0140-6736(96)90321-9 8602096

[22] Ephraim RKD Osakunor DNM Denkyira SW Eshun H Amoah S Anto EO Serum calcium and magnesium levels in women presenting with pre-eclampsia and pregnancy-induced hypertension: a case-control study in the Cape Coast metropolis, Ghana. BMC Pregnancy Childbirth. 2014;14:390. 10.1186/s12884-014-0390-2 25410280 PMC4243325

[23] Anya SE Seasonal variation in the risk and causes of maternal death in The Gambia: Malaria appears to be an important factor. Am J Trop Med Hyg. 2004;70:510-3. 10.4269/ajtmh.2004.70.510 15155982

[24] Dorman EK Shulman CE Kingdom J Bulmer JN Mwendwa J Peshu N Impaired uteroplacental blood flow in pregnancies complicated by falciparum malaria. Ultrasound Obstet Gynecol. 2002;19:165-70. 10.1046/j.0960-7692.2001.00545.x 11876809

