# Supplementary material for: The association of malaria infection and gestational hypertension in Africa: Systematic review and meta-analysis
Source: J Glob Health. 2020 Sep 8;10(2):020417. doi: 10.7189/jogh.10.020417 (PMC7568931; doi:10.7189/jogh.10.020417)
Supplement: Online Supplementary Document [file jogh-10-020417-s001.pdf]

Figure 3: PRISMA flow diagram of selected papers in the Systematic review.

Figure 3 shows the searched sources and identification process of the selected papers in this meta-analysis.

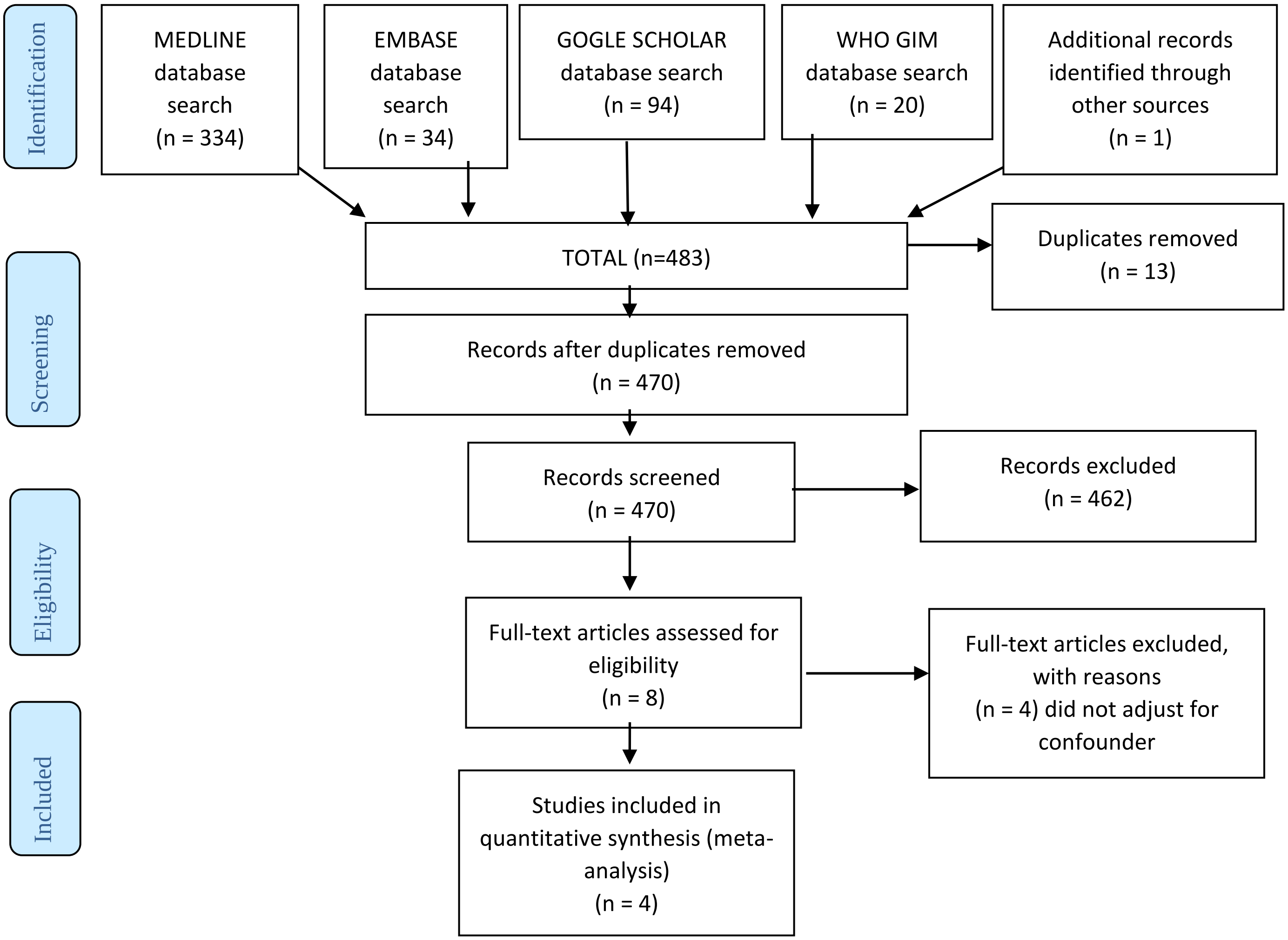

Table 1. Overview of the included studies.

The table shows the countries, sample size, study design, characteristics of participant and the results of the selected papers for the systematic review.

Country	Author and Year	Sample size	Participants characteristics	Results comparison
**Sudan**	(9)	143 Cases/143 control	Recruited case and controls from hospital, Excluded Twins and Diabetes.	Multivariate analysis adjusted for age, primigravidae, Hx of malaria, Family Hx of HT, BMI, Blood Gp, Placenta malaria, Education level, Lack of ANC
**Ghana**	(21)	120 Cases/160 control	Recruited case and control from hospital, excluded chronic HT, on Antihypertensive drugs, eclampsia, diabetes autoimmune, renal diseases.	Adjustment of confounder in the multivariate analysis: Age, gravidity, parity, BMI, contraceptive use, Abortion, New paternity, and Malaria for GH
**Senegal**	(8)	223 Cases/240 control	Recruited cases and control from hospital, matched them by age, parity and prematurity	Multivariate analysis, adjusted for placenta malaria, residence, parity, past pregnancies, ANC visit, family Hx of HT, period of delivery-seasonality, illiteracy, marital status.
**Senegal**	(Sartelet, 1996)	32 Cases/220 control	Recruited cases and control from hospital, were similar on mean age, no of previous pregnancies	Adjusted for age, no of previous pregnancies, twin delivery, maternity centre and date of delivery.

Table 2: Quality assessment using critical appraisal skills programme (CASP) checklist tool

Author, Year	***Focused question***	***Appropriate method to answer the question***	***Recruitment of cases***	***Recruitment of controls***	***Recruitment Overall***	***Exposure accurately measured***	***Confounding factors accounted for***	***Confounding factors taken into account in the design/analysis***	***What are the results?***	***How precise are the results?***	***Do you believe the results?***	***Can the results be applied to the local population?***	***Do the results of this study fit with other available evidence?***
**Adam I et al 2011**	Yes	Yes	Hospital based recruitment	Control selected from same hospital, some bias compared to exposure in community.	No sample size or power calculation. Case and control not comparable in their primigravidae status, family hx of HT [more % in cases], control lacked ANC care more than cases.	Exposure accurately measured, Giemsa stain of placental blood and placenta histology	Excluded Twins and Diabetes, known predictors.	Multivariate analysis adjusted for age, primigravidae, Hx of malaria, Family Hx of HT, BMI, Blood Gp, Placenta malaria, Education level, Lack of ANC	Multivariate analysis Family Hx of HT OR=5.7 95%CI [2.9-11.5]. Placental malaria OR=2.3 95%CI [1.0-5.2]	Results are precise, P=0.04 for placental malaria, moderate strength of the association OR 95%CI 1.0-5.2. Just above 1.	Yes	Internal validity Yes.	Generalizability, yes results are comparable with previous studies in Africa.

Author, Year	***Focused question***	***Appropriate method to answer the question***	***Recruitment of cases***	***Recruitment of controls***	***Recruitment Overall***	***Exposure accurately measured***	***Confounding factors accounted for***	***Confounding factors taken into account in the design/analysis***	***What are the results?***	***How precise are the results?***	***Do you believe the results?***	***Can the results be applied to the local population?***	***Do the results of this study fit with other available evidence?***
**Ephraim R et al 2014**	Yes	Yes, case control	Multicentre hospital-based recruitment. Hypertensive with or without proteinuria post 20wks	Age matched controls normal tensive and without proteinuria post 20wks.	No sample size calculated prior the study	Malaria dx not explained	Adjustment of confounder in the multivariate analysis, Age, gravidity, parity, BMI, contraceptive use, Abortion, New paternity and Malaria for GH not PE	excluded chronic HT, on Antihypertensive drugs, eclampsia, diabetes autoimmune, renal diseases.	malaria not associated with GH, OR=2.92 95%CI [0.5 to 17.15]	Non-significant and wide CI, possibly insufficient power.		Internal validity cannot be ascertained, no number of malaria exposed case and control	

Author, Year	***Focused question***	***Appropriate method to answer the question***	***Recruitment of cases***	***Recruitment of controls***	***Recruitment Overall***	***Exposure accurately measured***	***Confounding factors accounted for***	***Confounding factors taken into account in the design/analysis***	***What are the results?***	***How precise are the results?***	***Do you believe the results?***	***Can the results be applied to the local population?***	***Do the results of this study fit with other available evidence?***
**Sartelet H et al 1996**	Association of pre-eclampsia and placenta malaria?	Yes, case control design	Hospital based two centres. No exclusion of participants. Did not use the current definition of pre-eclampsia, BP and proteinuria.	controls similar to cases, when compared on mean age and no of previous pregnancy.	No sample size precalculated,	placenta malaria accurately measured. Outcome measured in old standards, diastolic BP only. No proteinuria.	Adjusted for age, no of previous pregnancies, twin delivery, maternity centre and date of delivery.	Adjusting for date of delivery/seasonality is unique and valuable in malaria hypo endemic area	Multivariate odds ratio 3.3 95%CI [1.1 to 9.5.]	Significant results with moderate CI, possibly due to low power after adjusting multiple variables. The univariate CI was narrow.3.0 95%CI [1.3-6.9].	Yes, case control design	Internal validity Yes, Generalizability Yes.	Yes, a pioneer study.

Author , Year	***Focused question***	***Appropriate method to answer the question***	***Recruitment of cases***	***Recruitment of controls***	***Recruitment Overall***	***Exposure accurately measured***	***Confounding factors accounted for***	***Confounding factors taken into account in the design/analysis***	***What are the results?***	***How precise are the results?***	***Do you believe the results?***	***Can the results be applied to the local population?***	***Do the results of this study fit with other available evidence?***
**Ndao C T et al 2009**	Relationship between placental malaria and hypertension during pregnancy.	case control design	Hospital based study	Control matched to cases, age parity and prematurity	case and control were matched for Age, parity and date of delivery(prematurity). But did not do a matched analysis, rather unconditioned logistic reg. 69% drop in recruiting eligible cases/control due to data missing but suggested to be missing completely at random.	Placenta malaria accurately measured. Also, the outcome well categorised pre-eclampsia, eclampsia, gestational hypertension.	Compared case and control on many aspects, they were comparable: Insecticide treated bed nets use, quinine prophylaxis use by urine test, residence, age, religion, period of delivery-seasonality.	Variables included in the analysis, placenta malaria, residence, parity, past pregnancies, ANC visit, family Hx of HT, period of delivery-seasonality, illiteracy, marital status.	Multivariate OR=2.7 95%CI [1.0-7.6]	narrow CI, hence, results can be trusted.	Yes	Internal validity Yes, there may be variabilities across different endemicity regions.	Generalizable results, and in agreement with other studies.
